# Increased variability of fetal heart rate during labour: a review of preclinical and clinical studies

**DOI:** 10.1111/1471-0528.17234

**Published:** 2022-06-05

**Authors:** Mikko J. Tarvonen, Christopher A. Lear, Sture Andersson, Alistair J. Gunn, Kari A. Teramo

**Affiliations:** ^1^ Department of Obstetrics and Gynaecology University of Helsinki, and Helsinki University Hospital Helsinki Finland; ^2^ Fetal Physiology and Neuroscience Group Department of Physiology University of Auckland Auckland New Zealand; ^3^ Children’s Hospital, Paediatric Research Centre University of Helsinki, and Helsinki University Hospital Helsinki Finland

**Keywords:** acidaemia, cardiotocography, electronic fetal monitoring, fetal heart rate, increased variability, marked variability, neonatal morbidity, pregnancy, saltatory pattern, ZigZag pattern

## Abstract

Increased fetal heart rate variability (FHRV) in intrapartum cardiotocographic recording has been variably defined and poorly understood, limiting its clinical utility. Both preclinical (animal) and clinical (human) evidence support that increased FHRV is observed in the early stage of intrapartum fetal hypoxaemia but can also be observed in a subset of fetuses during the preterminal stage of repeated hypoxaemia. This review of available evidence provides data and expert opinion on the pathophysiology of increased FHRV, its clinical significance and a stepwise approach regarding the management of this pattern, and propose recommendations for standardisation of related terminology.

## Tweetable abstract

Increased fetal heart rate variability is parasympathetic‐mediated and is caused by acutely deteriorating placental function.

## 1 INTRODUCTION

Cardiotocographic (CTG) electronic fetal heart rate (FHR) monitoring is the gold standard for assessing fetal wellbeing during labour, although it has a very poor positive predictive value for fetal hypoxic–ischemic neural injury.^1^, ^2^, ^3^, ^4^, ^5^ In recent years, efforts have been made to improve the accuracy of fetal monitoring and the evaluation of intrapartum adaptation by emphasising that an adequate interpretation of a CTG tracing relies not only on recognition of FHR patterns but also on understanding the fetal physiology behind the patterns.^6^, ^7^, ^8^, ^9^, ^10^, ^11^

The evaluation of FHR patterns is based on baseline FHR, and the depth, duration, timing and frequency of FHR decelerations and associated changes in FHR variability (FHRV).^12^, ^13^, ^14^, ^15^, ^16^ Moderate levels of FHRV are associated with a well‐oxygenated fetus, whereas reduced or absent FHRV is a warning sign of fetal compromise.^17^, ^18^, ^19^, ^20^ Intriguingly, there is growing evidence suggesting that increased FHRV, characterised by high‐amplitude oscillations of FHR, may be important.^21^, ^22^ Experimental studies in fetal sheep have demonstrated that fetal compromise can be associated with transiently increased FHRV.^23^, ^24^, ^25^ Recently, on the basis of intrapartal visual evaluation, increased FHRV has been associated with increased risk of fetal acidaemia at birth and early neonatal complications in human labour.^26^, ^27^, ^28^, ^29^, ^30^ The definition and classification of increased FHRV vary in the literature and in CTG monitoring guidelines.^29^, ^31^, ^32^, ^33^ Furthermore, the pathophysiologic changes that mediate increased FHRV during labour remain poorly understood.^34^, ^35^, ^36^, ^37^, ^38^ There is a lack of consensus on factors associated with increased FHRV during labour.^27^, ^28^, ^29^, ^30^, ^39^, ^40^, ^41^, ^42^ Moreover, the clinical significance of increased FHRV is uncertain.^27^, ^43^, ^44^

The aims of the present review are to delineate the pathophysiology of increased FHRV, clarify the related terminology, and elucidate its potential clinical utility. We further propose that broadly there exist two hypoxaemia‐related patterns of increased FHRV during labour: a pattern which has variously been called the ‘ZigZag’ or ‘saltatory’ pattern and is more often observed earlier in labour and not associated with deep repetitive FHR decelerations; and secondly, a pattern of increased FHRV observed in association with deep FHR decelerations in late labour. Suppression of FHRV, particularly in the presence of deep decelerations, remains an ominous sign that requires clinical attention. Nonetheless, it is increasingly being understood that this is not a universal finding in fetuses at risk of intrapartum acidaemia and hypoxic–ischemic injury.^45^, ^46^, ^47^, ^48^ We therefore believe that a simplified definition and classification of these two patterns of increased FHRV will help to increase awareness and to alert birth attendants.

## 2 FETAL ADAPTATION DURING LABOUR

During childbirth, uterine contractions result in repeated, brief reductions in uteroplacental perfusion, causing intermittent relative fetal and placental relative hypoxaemia. This reduction is associated with a transient fall in blood pH, base excess (BE) and oxygen tension, and a rise in carbon dioxide and base deficit (BD), even in normal, uncomplicated labour.^49^, ^50^, ^51^, ^52^ The fetus compensates for moderate to severe intrapartum stress by activating the peripheral chemoreflex, leading first to FHR decelerations, presumptively to reduce myocardial oxygen demand, and second to trigger peripheral vasoconstriction preferentially to support blood flow to the heart, brain and adrenal glands.^11^, ^53^, ^54^, ^55^, ^56^, ^57^ A healthy term fetus with a normally developed and functioning placenta is able to adapt to the typical frequency and intensity of uterine contractions without adverse consequences.^58^ However, if the interval between the contractions is too short, or placental function is compromised, prolonged impairment of oxygen delivery may lead to tissue hypoxia, metabolic acidaemia, and persistent reduction in fetal cerebral oxygenation.^59^, ^60^, ^61^, ^62^, ^63^, ^64^ If these episodes of hypoxaemia continue, fetal cardiac output is progressively compromised, leading to fetal hypotension and hypoperfusion, potentially resulting in hypoxic–ischemic brain injury.^65^, ^66^, ^67^, ^68^, ^69^, ^70^, ^71^ The progressive worsening intrapartum fetal hypoxaemia can be observed as changes in baseline FHR and deeper FHR decelerations,^6^, ^72^, ^73^ but once deeper decelerations are established there is typically little further change in FHR.^74^ Increased FHRV typically develops in the early stage of fetal hypoxia^23^, ^29^, ^36^, ^43^ but can be seen also in FHR tracings of fetuses during the preterminal stage of repeated asphyxia (Appendix S1).^21^, ^35^, ^70^

## 3 PATHOPHYSIOLOGY OF INCREASED FHRV: INSIGHT FROM PRECLINICAL ANIMAL STUDIES

Increased FHRV patterns are seen rarely in antenatal FHR tracings, occurring almost exclusively during the active stage of labour.^75^, ^76^ This suggests that labour‐induced fetal stress, i.e. intermittent gas exchange disruption and consequent fetal hypoxaemia caused by intense uterine contractions, contributes to the intrapartum increased FHRV pattern.^29^, ^77^ Many studies in chronically instrumented fetal animals have used simulated intrapartum stress to improve clinical understanding of compensation mechanisms in the human fetus during birth, as well as the accompanying changes in FHR and FHRV.^47^, ^78^, ^79^, ^80^, ^81^, ^82^, ^83^ Animal studies can be broadly separated into those that study sustained periods of hypoxaemia and those that study intermittent periods of repeated hypoxaemia. The latter is more characteristic of the repetitive nature of hypoxaemia during intrapartum uterine contractions. Sustained periods of hypoxaemia can occur for example during sentinel events (e.g. placental abruption, cord prolapse, uterine rupture) but here we propose that sustained periods of mild hypoxaemia may have an underappreciated role in some instances of increased FHRV.

### 3.1 Sustained hypoxaemia

In 1977, Dalton et al.^78^ reported increased FHRV during sustained moderate hypoxaemia in fetal sheep achieved by maternal inhalation of decreased oxygen, an observation that has been replicated multiple times.^43^, ^84^, ^85^ This is typically observed in the presence of bradycardia; for example in the study by Parer et al.^43^ FHR fell from 170 ± 22 to 139 ± 21 bpm after 5 minutes of hypoxaemia with a fall in mean pO_2_ from 20.7 to 11.3 mmHg.^43^ Likewise, more severe hypoxaemia (mean pO_2_ from 22.4 to 5.8 mmHg) induced by complete umbilical cord occlusion (UCO) results in increased FHRV during the early minutes of UCO in association with marked bradycardia.^47^, ^86^ Of particular interest, mild hypoxaemia in fetal sheep is associated with increased FHRV without a marked fall in FHR.^43^ Similarly, mild hypoxaemia in fetal monkeys was associated with an average fall in FHR from 199 to 178 bpm.^87^, ^88^ However, it is notable that individual fetuses that show a less pronounced fall in pO_2_ displayed increased FHRV without a fall in FHR. In those that had a fall in FHR, this was often preceded by increased FHRV.^88^

### 3.2 Repeated brief hypoxaemia

Repeated partial or complete UCOs have been used in fetal sheep to simulate the repetitive nature of hypoxaemia induced by uterine contractions. Each UCO is associated with a FHR deceleration, with more severe UCOs associated with deeper decelerations.^74^, ^89^, ^90^ These studies have shown that the early stages of fetal adaptation to repetitive brief hypoxaemia are associated with increased FHRV between FHR decelerations.^23^, ^25^, ^91^ When UCO continues, the initial increase in FHRV diminishes and FHRV returns to baseline values. The terminal phase of UCO resulting in cardiovascular compromise and hypotension is associated with variable FHRV patterns.^92^ In the study by Westgate et al.^23^ in fetal sheep, two‐thirds developed mild suppression of FHRV, with the remaining third showing a marked increase in FHRV. The mechanism underlying the differing FHRV patterns remains unknown.

### 3.3 Autonomic origin

During the prepartum period, in a healthy normoxic fetus, FHRV is complexly and constantly regulated by both the sympathetic and parasympathetic nervous systems,^78^, ^79^ which are integrated at the sinoatrial node in concurrence with its own inborn rhythm.^37^, ^38^, ^93^ Over the past decades, the pathophysiology of increased FHRV during labour has been explained by the hypothesis that during rapid hypoxaemia the fetus has insufficient time to release catecholamines, leading to impaired central organ perfusion, and a magnified autonomic response caused by instability of sympathetic and parasympathetic nervous systems.^27^, ^36^, ^79^, ^84^, ^94^, ^95^

In contrast, more recent studies in fetal sheep have employed multiple forms of autonomic blockade during repeated UCOs to illustrate that FHRV during labour (once repetitive decelerations are apparent) is solely mediated by the parasympathetic nervous system, as recently reviewed.^96^ For example, neither complete β‐adrenergic blockade with propranolol^83^, ^97^, ^98^ nor chemical sympathectomy with 6‐hydroxydopamine neurotoxin^37^, ^99^ reduced FHRV during repeated UCOs. In contrast, FHRV was abolished with parasympathetic blockade with either atropine sulphate or bilateral vagotomy.^38^ Likewise, during sustained periods of moderate fetal hypoxaemia induced by maternal hypoxaemia, atropine but not propranolol prevented the increase in FHRV.^43^, ^89^ The mechanisms underlying the shift from dual sympathetic and parasympathetic control of FHRV during normoxia to parasympathetic dominance during both repetitive and sustained hypoxaemia are unknown but may involve feedback inhibition from high circulating catecholamine concentrations.^96^ Increased FHRV during both sustained and intermittent hypoxaemia is therefore likely mediated by increased parasympathetic activity, although the upstream mechanisms driving increased parasympathetic activity are likely distinct in each scenario.

## 4 HUMAN STUDIES

### 4.1 Definitions and incidences

Periods of increased or high‐amplitude FHRV that are occasionally observed in routine intrapartum FHR recordings have been referred to by multiple terms over the years. Initially, these patterns were referred to as ‘marked irregularity’ by Hon and Lee,^100^ ‘rapid baseline fluctuations’ by Caldeyro‐Barcia et al.^101^ and ‘high‐amplitude oscillations’ by Hammacher et al.^102^The current literature includes descriptions of ‘marked variability’, the saltatory pattern and the ZigZag pattern. Table 1 gives a summary of the terminology, definitions and incidences of increased FHRV used by current human studies and clinical guidelines.

**TABLE 1:** Definition of increased variability patterns in the FIGO, RCOG and ACOG cardiotocography interpretation guidelines and related studies

CTG guideline and study	Increased FHRV pattern	Definition	Classification	Reference on which definition and classification are based	Incidence (%)
FIGO (2015)^32^	Saltatory pattern	A bandwidth value >25 bpm	Pathological if >30 min	Nunes et al. (2014)^95^: four cases with a prolonged saltatory pattern lasting for >20 min during the last 30 min before birth from databases of 13 859 CTG tracings	0.03
RCOG (NICE) (2017)^33^	Saltatory feature	Baseline variability amplitude range > 25 bpm	Non‐reassuring if 15–25 min. Abnormal if >25 min	NICE (2017)^33^: The Guideline Committee decided that the time cut‐off between the non‐reassuring and abnormal categories would be 25 bpm for >25 min because it is easy for clinicians to remember. The ≥15 min was introduced to avoid unnecessary interventions based on the presence of a single feature	
ACOG (NICHD) (2010)^129^	Marked variability	Amplitude range >25 bpm	No required duration mentioned. Analysed in 10‐min epochs. Category II pattern in 3‐tiered classification of FHR abnormalities	NICHD (1997)^130^: electronic fetal heart rate monitoring: research guidelines for interpretation. National Institute of Child Health and Human Development Research Planning Workshop	
Cibils (1976)^144^	Saltatory pattern	Variability of >25 bpm	No required duration mentioned	Analysis of 1304 CTG tracings.^144^ FHR oscillation frequencies by Hammacher et al.^102^	7.8
O’Brien‐Abel & Benedetti (1992)^44^	Saltatory pattern	Amplitude changes of >25 bpm	Oscillatory frequency of >6/min for ≥1 min	Analysis of 433 CTG tracings.^44^ Not specified on which the definition is based	2.3
Polnaszek et al. (2020)^27^	Marked variability	Fluctuations in FHR amplitude of >25 beats bpm based on 10‐min epochs	No required duration mentioned	Analysis of 8580 CTG tracings.^27^ For the definition, see ACOG guideline.^129^	4.5
Gracia‐Perez‐Bonfils et al. (2021)^28^	ZigZag pattern	Increased bandwidth of the FHR baseline (>25 bpm)	From 1 to 30 min. Differs from the saltatory pattern also in uniformity of the trace	Analysis of 500 CTG tracings.^28^	≥1 min in 30.1 ≥2 min in 8.9
Tarvonen et al. (2021)^29^, ^30^, ^41^, ^42^	ZigZag pattern	FHR baseline amplitude changes of >25 bpm	≥2 min	Analysis of 245 CTG tracings and cord blood EPO measurements.^41^ Analysis of 4988 term CTG tracings.^29^ Analysis of 5150 preterm, term and post‐term CTG tracings.^30^, ^42^	13.1 11.7 11.3

Abbreviations: ACOG, The American College of Obstetricians and Gynecologists; CTG, cardiotocography; EPO, erythropoietin; FHR, fetal heart rate; FIGO, The International Federation of Gynaecology and Obstetrics; NICE, National Institute for Health and Care Excellence; NICHD, *Eunice Kennedy Shriver* National Institute of Child Health and Human Development; RCOG, The Royal College of Obstetricians and Gynaecologists.

Although the saltatory pattern is well known, it is notable that in a recent study of a large obstetric cohort, only six (1.0%) of the 582 CTG recordings showed increased FHRV; the duration of a single increased FHRV episode lasted between 15 and 25 minutes, and in one (0.2%) case was >25 minutes (28 minutes).^29^ Furthermore, not a single increased FHRV pattern with a duration of >30 minutes was found in the cohort of 5150 childbirths.^29^, ^30^ These findings are in agreement with suggestion that the saltatory pattern, as defined by FIGO and NICE,^32^, ^33^ is almost nonexistent.^28^, ^29^

### 4.2 Association with fetal acidaemia and compromise

A recent study including 8580 births by Polnaszek et al. showed that marked variability patterns occurred in 6.7% of the 149 cases with cord blood acidaemia (UA pH <7.10).^27^ Marked variability was associated with an increased risk of elevated cord blood lactate and an increased risk of respiratory distress, although no association with composite neonatal morbidity was found.^27^ In their study, episodes of marked variability were most common during the final 10 minutes prior to birth, becoming progressively less common in the 2 hours studied prior to birth. The authors concluded that marked FHRV in isolation does not predict neonatal acidaemia.^27^ This is in agreement with the study by O’Brien‐Abel and Benedetti, who concluded that a pattern of increased FHRV can be considered benign when observed in the absence of other abnormal periodic FHR changes, and in the presence of normal FHR variability before and after the high‐amplitude oscillations of FHR.^44^ Another recent study of 1070 fetuses who had fetal scalp blood sampled during labour showed that increased fetal scalp blood lactate level was associated with increased short‐term FHRV.^103^ The association was observed in all four 30‐minute epochs during the last 2 hours prior to birth.^103^ These findings support the concept that the early stages of intrapartum fetal hypoxaemia is associated with increased FHRV.

Recently, Tarvonen et al.^41^ investigated the episodes of increased FHRV ≥2 minutes in duration (the ZigZag pattern) in a retrospective study of 194 CTG tracings of fetuses with low Apgar scores and their 51 healthy controls. The ZigZag pattern was associated with both cord blood acidaemia and high concentrations of cord blood erythropoietin (EPO) at birth.^41^ Fetal hypoxaemia strongly stimulates EPO synthesis,^104^, ^105^, ^106^, ^107^ and hence high plasma EPO concentration is a marker of the severity of fetal hypoxaemia.^108^, ^109^

Further work by Tarvonen et al.^29^ has highlighted the association between the ZigZag pattern and FHR decelerations. The presence of increased FHRV or late decelerations, or both, in the CTG recordings during the last 2 hours of labour has been shown to increase by three‐fold the likelihood of severe hypoxaemia‐related complications (i.e. UA pH <7.10 and/or BE < −12.0 mEq/l and/or 5‐minute Apgar score <4 and/or intubation for resuscitation and/or grade II/III neonatal encephalopathy) in newborn infants. A CTG recording with both ZigZag pattern and late decelerations occurred in 76.9% (123/160) of cases with severe neonatal complications but in only 5.6% (201/3620) of cases with no complications.^29^ Strikingly, in the vast majority of cases, a rapid transition was observed from an initially ‘normal’ or ‘reassuring’ FHR trace without decelerations, to the pattern of increased FHRV and the subsequent appearance of late decelerations. The median time between the end of the first ZigZag episode and the onset of late decelerations was 9 minutes.^29^ Two previous case reports, and one study with a population of high‐risk patients, further support the concept that the concurrent occurrence of increased FHRV and late decelerations indicates an increased risk of severe hypoxaemia.^34^, ^35^, ^110^

### 4.3 Risk factors

Observations in human fetuses suggest that maternal and fetal background factors may play a role in the origin and development of intrapartum increased FHRV. Among a cohort of 5150 childbirths, the ZigZag pattern only occurred in term and post‐term pregnancies,^42^ with an increasing incidence with advancing gestation.^29^, ^30^, ^41^, ^42^ This finding confirms a previous observation that the presence of increased FHRV is rare in preterm fetuses.^44^ These observations are in agreement with a study in which umbilical cord plasma EPO levels increased significantly after 40 weeks of gestation in pregnancies with spontaneous onset of labour,^111^ suggesting that placental insufficiency after 40 weeks of gestation may contribute to the occurrence of the hypoxaemia‐related increased FHRV.

Recently, male sex of the fetus, nulliparous pregnancy and post‐term pregnancy of ≥42 weeks were independently associated with the ZigZag pattern.^42^ Consequently, the presence of any of these three independent risk factors, or a combination of them, increased the likelihood of the occurrence of increased FHRV up to 16‐fold.^42^ Another recent study showed that fetuses of women with gestational diabetes mellitus (GDM) were more likely to have the ZigZag pattern than in pregnancies of women with no GDM.^30^ Moreover, fetuses of GDM mothers with two abnormal oral glucose tolerance test values had the strongest association with the intrapartal ZigZag pattern.^30^ These findings are in agreement with previous studies in GDM pregnancies, in which impaired glucose metabolism diagnosed in early pregnancy, as a result of more severe form of GDM, is associated with both functional and structural placental changes.^112^, ^113^, ^114^, ^115^

It is further worth appreciating that both maternal and fetal infections have been previously linked with increased FHRV. In preterm fetal sheep, acute exposure to high‐dose lipopolysaccharide (a bacterial cell wall component that induces a systemic inflammatory response) triggered an increase in FHRV between 2 and 4 hours after exposure.^116^, ^117^ This increase in FHRV, however, is only observed after an acute inflammatory stimulus that triggers a rapid inflammatory and cardiovascular response, whereas a stable inflammation response has little effect on FHRV even if prolonged.^118^ In two recent studies, 4% (nine of 224)^119^ and 33% (four of 12)^120^ of human fetuses of COVID‐19 parturients showed the ZigZag pattern in CTG tracing. Similarly, chorioamnionitis and funisitis have been associated with the ZigZag pattern during labour.^121^, ^122^, ^123^ It remains unclear whether these human findings are due to infection/inflammation exacerbating hypoxaemia during labour or are an independent effect of infection/inflammation increasing FHRV. Unfortunately, the effect of infection/inflammation on FHRV during labour‐like hypoxaemia has not been studied in animals.

### 4.4 Nomenclature

Increased FHRV is defined as baseline amplitude changes of >25 bpm.^32^, ^33^, ^124^ Based on recent findings, even short episodes of ≥1 minute of increased FHRV are associated with unfavourable fetal and neonatal outcomes.^28^, ^29^, ^30^, ^41^ Nonetheless, confusion about terminology used to describe increased FHRV patterns has been a longstanding problem. It is well known that standardised terminology to describe intrapartum CTG may avoid miscommunication among clinicians caring for parturients and can improve the safety of childbirth.^31^, ^124^, ^125^, ^126^ Furthermore, unified terms when evaluating whether FHR patterns are reassuring or nonreassuring (i.e. normal, suspicious or pathological) help providers to decide when to intervene.^124^, ^125^, ^126^, ^127^, ^128^ Currently, according to FIGO, RCOG and ACOG CTG guidelines^31^, ^32^, ^33^, ^127^, ^128^, ^129^, ^130^ and the wider literature,^27^, ^28^, ^42^, ^44^ a number of different terms of increased FHRV patterns are used that actually describe the same FHRV phenomenon. We therefore propose that terms such as ‘saltatory pattern’, ‘ZigZag pattern’ and ‘marked variability’ should be abandoned, and the common term ‘increased variability’ should be used in clinical guidelines in the same way the term ‘reduced variability’ already reflects decreased levels of FHRV.

## 5 LINKING THE PRECLINICAL AND CLINICAL FINDINGS

**FIGURE 1:**
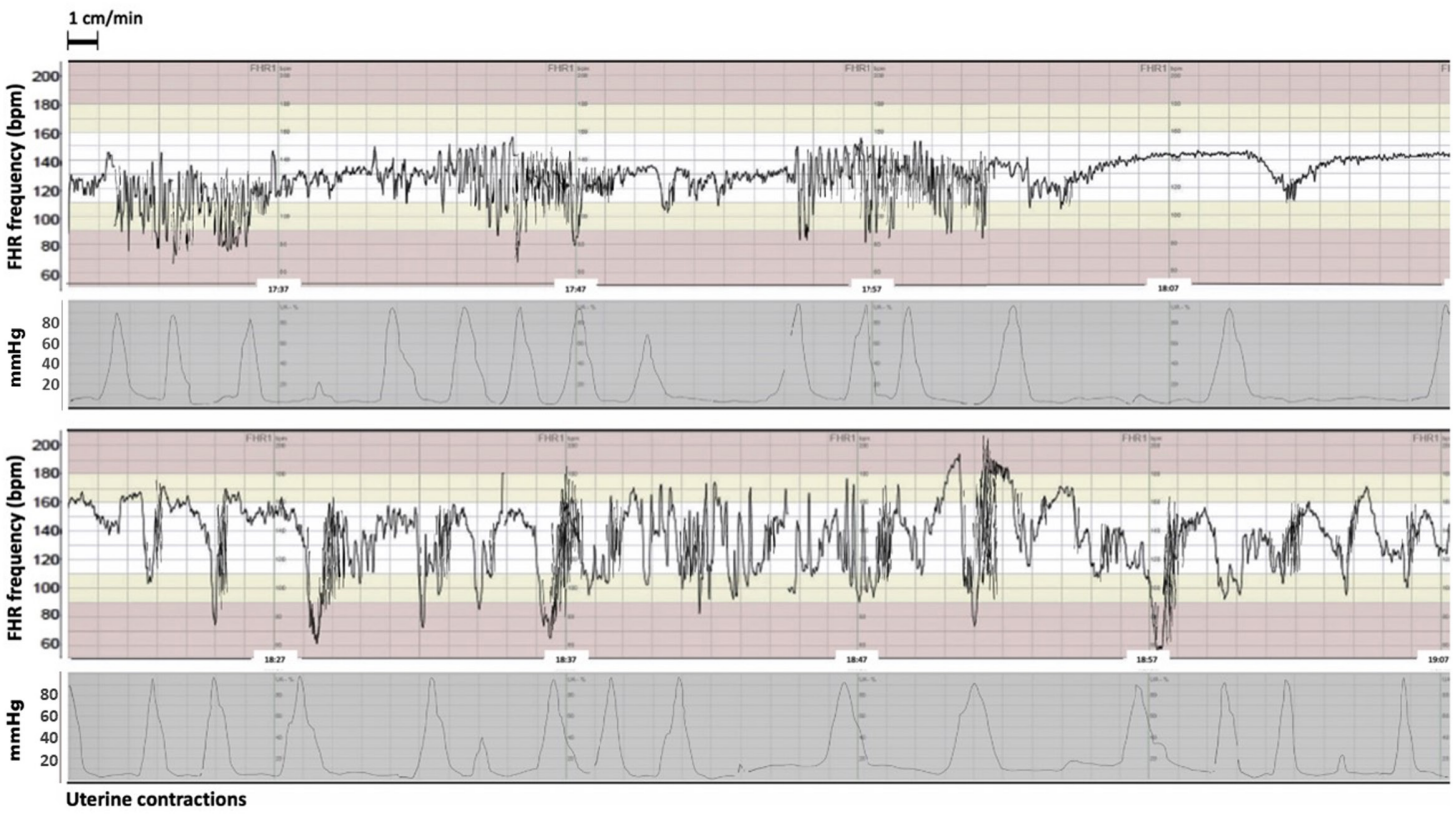
Intrapartum CTG recording at 41^+0^ weeks of pregnancy. Upper trace: Normal baseline FHR (135 bpm) followed by three increased FHRV episodes with durations of 6–7 minutes. Note the varying frequency of uterine contraction, with the periods of increased FHRV associated with greater contraction frequency, with resolution during periods of reduced contraction frequency. These changes are further followed by increased FHR, reduced FHRV and late decelerations. Lower trace: The same fetus with repeated interdeceleration increased FHRV patterns followed by unstable baseline FHR. A male fetus was born vaginally spontaneously at the end of the tracing. Umbilical cord blood gas analysis at birth showed deep acidaemia, UA pH 6.95, UA BE −15.4 and UV pH 7.04, UV BE −12.1. FHR was recorded via fetal scalp electrode with paper speed 1 cm/min. BE, base excess; bpm, beats per minute; CTG, cardiotocography; FHR, fetal heart rate; FHRV, fetal heart rate variability; UA, umbilical artery; UV, umbilical vein

Collectively the evidence from human labour suggests that increased FHRV broadly occurs in two situations: (1) in association with repetitive FHR decelerations, manifesting as brief periods of increased FHRV mainly between decelerations, and (2) increased FHRV without repetitive FHR decelerations. This second pattern is more typically observed earlier during labour and manifests as a relatively longer period of increased FHRV.^29^, ^41^ Figure 1 shows typical examples of these two patterns in intrapartum CTG recordings. In this section we attempt to parallel human observations with insight from preclinical animal studies in order to explain the pathophysiological origins of FHRV.

### 5.1 Increased FHRV with concurrent decelerations

The occurrence of increased FHRV associated with FHR decelerations appears to match the scenario described above in the ‘repeated brief hypoxaemia’ section. These animal studies illustrate that FHRV increases firstly during the early phase of adaptation to repetitive brief hypoxaemia, and therefore increased FHRV can be expected to occur early after the first appearance of frequent FHR decelerations during labour. During prolonged exposure to intense repetitive brief hypoxaemia, fetal cardiovascular adaptation can progressively fail in association with worsening acidaemia. Animal evidence suggests that this late phase can be associated with either increased or reduced FHRV.^23^ Increased FHRV can sometimes be observed during the nadir of repetitive brief decelerations, but there is little understanding about whether this has any different implications than increased FHRV between decelerations.

The human findings that increased FHRV, particularly towards the end of labour, is associated with increased risk of fetal acidaemia or compromise is in keeping with recent evidence that suppression of FHRV is less predictive of fetal acidaemia than previously thought.^45^, ^46^, ^47^, ^48^ Fetal compromise resulting from repetitive brief hypoxaemia episodes appear to be associated with either ‘abnormally’ increased or suppressed FHRV. These alternate patterns appear to be related to different patterns of parasympathetic activity during fetal compromise, the mechanisms of which are still imperfectly understood.^96^

### 5.2 Increased FHRV without concurrent decelerations

The pattern of increased FHRV during labour observed without FHR decelerations (including the patterns previously called saltatory or ZigZag patterns) has remained poorly understood. This pattern is more commonly observed in early labour. In considering the potential pathophysiological mechanisms, the following observations are important:

- The absence of FHR decelerations suggests the absence of significant repetitive brief hypoxaemia episodes.
- The pattern is observed in the presence of either a stable baseline FHR or on top of a modest fall in baseline FHR (although this may be obscured by increased FHRV).
- The duration of these patterns is longer than a typical uterine contraction.
- The majority of risk factors associated with the pattern are equally risk factors for impaired placental function, including severe form of GDM, post‐term pregnancy, elevated fetal cord blood EPO concentration, high placental weight relative to birthweight, and the occurrence of late decelerations.

We propose that this pattern represents a comparatively mild but more prolonged hypoxaemia than the pattern observed in association with repetitive variable FHR decelerations, consistent with animal experiments that have modelled ‘sustained hypoxaemia’. Indeed, there is evidence in both fetal sheep and monkeys that mild hypoxaemia can trigger a parasympathetic‐mediated increase in FHRV either in the absence of a fall in FHR, or with only a modest fall in FHR.^21^, ^37^, ^84^ The fact that these patterns are frequently not synchronised with uterine contractions, suggests that they reflect an acute deterioration of placental function leading to a relatively prolonged period of mild fetal hypoxaemia. Supporting this, in the recent studies by Tarvonen et al.^29^, ^42^ a close temporal association was found between an initially ‘normal’ or ‘reassuring’ FHR trace without decelerations, the appearance of the pattern of increased FHRV and the subsequent appearance of late decelerations. This rapid appearance of FHR decelerations suggests a deterioration of placental reserve or uteroplacental gas exchange.

Alternatively, and considering the association of the increased FHRV with GDM, the fetus is an obligate user of glucose and thus increases oxygen consumption during acute hyperglycaemia which can lead to hypoxaemia and acidaemia.^131^, ^132^, ^133^ An acute maternal hyperglycaemia during labour may therefore exacerbate labour‐induced hypoxaemia and contribute to the pattern of increased FHRV. Consistent with this speculation, GDM is associated with an increased risk of fetal hypoxaemia and acidaemia in labour.^30^, ^134^, ^135^, ^136^

It is further notable that greater duration of a single increased FHRV episode was associated with higher risk of fetal compromise: a mean duration of 4.8 minutes was associated with no neonatal complications, 6.5 minutes was associated with moderate complications, and 10.7 minutes was associated with severe complications.^29^ It is important to note that this pattern of increased FHRV predominantly occurs during early labour but is associated with later outcomes at birth. Based on these observations, we propose that the pattern of increased FHRV represents mild hypoxaemia, which is not a significant threat to the fetal wellbeing but identifies a fetus with impaired uteroplacental function that is at increased risk of failure to adapt to the challenge of repetitive hypoxaemia during labour. Supporting this concept, Table 2 shows that among a cohort of term fetuses,^29^ a high placental weight to birthweight ratio was associated with higher rates of increased FHRV during labour. It has been previously reported that placental enlargement may be an indicator of chronic fetoplacental hypoxaemia and is associated with increased risk of fetal compromise.^112^, ^113^ The association of higher risk of complications with longer durations of increased FHRV likely reflects more prolonged hypoxaemia and may reflect more significant impairment of placental function.

**TABLE 2:** Odds ratios (ORs) with 95% confidence interval (CI) for occurrence of increased FHRV pattern in CTG recording according to quartiles of placental weight to birthweight ratio in 4988 term deliveries

Quartiles of placental weight to birthweight ratio	Increased FHRV	Increased FHRV	Crude OR	95% CI	Adjusted^a^ OR	95% CI
	Present (*n* = 582)	Absent (*n* = 4406)				
1st	110	1102	Reference		Reference	
2nd	121	1101	1.10	0.84–1.44	1.05	0.80–1.41
3rd	155	1103	1.41	1.09–1.82	1.40	1.07–1.80
4th	196	1100	1.79	1.39–2.29	1.77	1.37–2.27

*Note*: The ORs and 95% CIs for the increased FHRV pattern were estimated by fitting logistic regression models. The logistic regression analysis was performed using R version 3.6.0.

Abbreviations: CI, confidence interval; CTG, cardiotocography; FHRV, fetal heart rate variability; OR, odds ratio.

^a^ Adjusted for parity, gestational age at delivery, maternal age ≥35 years, gestational diabetes mellitus, pre‐eclampsia, maternal fever ≥38.0°C, smoking, fetal sex and birthweight *z*‐score.

## 6 CLINICAL CONSIDERATIONS

The need for research of increased FHRV in conjunction with other FHR patterns has been called for in previous papers.^27^, ^137^, ^138^ Based on the cohort of 4988 term deliveries including 160 cases with hypoxaemia‐related fetal and neonatal complications,^29^ we estimated the number of caesarean deliveries that need to be performed (NNT) to prevent one case of cord blood acidaemia or neonatal hypoxaemia‐related morbidity (Table 3).^139^ In the setting of the combined occurrence of increased FHRV pattern followed by repetitive late decelerations at 120–90 minutes before birth, the NNT was suggested at four, which is relatively low (Table 3).^139^ However, when evaluated over the last 2 hours (120–0 minutes) of labour, the NNT was nine (Table 3). Similarly, the combined occurrence of increased FHRV and late decelerations at 120–90 minutes before birth had an adjusted odds ratio (aOR) of 33.0, and 120–0 minutes before birth an aOR of 5.4, for hypoxaemia‐related morbidity when compared with cases without these FHR patterns.^29^ Hypothetically, these findings may reflect a longer exposure time to intrapartum hypoxaemia in fetuses showing the pattern of increased FHRV and late decelerations occurring at or earlier than 90 minutes before birth compared with fetuses showing the same pattern FHR patterns for the first time immediately before birth. These NNTs are comparable to those which Cahill et al.^46^ have reported concerning FHR deceleration area, and deceleration area combined with fetal tachycardia, as discriminatory for fetal acidaemia and neonatal morbidity (NNTs five and six, respectively).

**TABLE 3:** Estimated number of caesarean deliveries needed to be performed for the CTG patterns predicting hypoxaemia‐related fetal and neonatal complications at 120–90 minutes and at 120–0 minutes before birth in term fetuses (*n* = 4988)

FHR pattern	Number (*n* = 4988)	NNT	Number (*n* = 4988)	NNT
		120–90 min before birth		120–0 min before birth
CTG with prolonged decelerations (with a duration of ≥3 min) and/or tachycardia episodes and/or reduced variability and/or uterine tachysystole but without increased FHRV pattern or late decelerations	3320 (66.6)	78.0	3851 (77.2)	60.1
CTG with late decelerations (increased FHRV pattern overlooked)	253 (5.1)	37.5	1934 (38.8)	49.4
CTG with increased FHRV pattern or late decelerations	214 (4.3)	23.3	1565 (31.4)	28.7
CTG with increased FHRV pattern or late decelerations or both	311 (6.2)	16.8	2096 (42.0)	21.0
CTG with increased FHRV pattern and late decelerations	97 (1.9)	3.9	531 (10.6)	9.0

*Note*: Data are presented as number (%). Increased FHRV pattern: FHR baseline amplitude changes of >25 bpm with a duration of ≥2 minutes. Hypoxaemia‐related fetal and neonatal complications: UA pH <7.10 and/or BE < −12.0 mEq/l and/or 5‐minute Apgar score <4 and/or intubation for resuscitation and/or grade II/III neonatal encephalopathy.

Abbreviations: CTG, cardiotocography; FHR, fetal heart rate; NNT, number needed to treat.^139^

Previously, Downs and Zlomke have suggested that a clinician should consider intrauterine resuscitation methods (Appendix S1) to improve the fetal environment *in utero* when an increased FHRV pattern occurs in isolation in an intrapartum CTG tracing.^140^ Indeed, in a recent study, the majority of NICHD Category II FHR tracings (in which category the increased FHRV pattern is included) were improved to normal Category I within 60 minutes of intrauterine resuscitation interventions.^141^

## 7 CONCLUSIONS

In recent studies with relatively large obstetric cohorts, the occurrence of increased FHRV in intrapartum CTG tracing has been associated with fetal acidaemia and a greater risk of neonatal complications. These studies have further associated increased FHRV with severe form of GDM, post‐term pregnancy, elevated fetal cord blood EPO concentration, high placental weight relative to birthweight, as well as the occurrence of late decelerations of FHR, all of which are associated with fetal hypoxaemia. Although caution is needed when extrapolating from animal studies, multiple parallels can be observed across species, suggesting a conserved FHRV response to hypoxaemia. In particular, we here present a new hypothesis to explain the pattern of increased FHRV that spans multiple contractions early in labour, and suggest that this mild hypoxaemia may represent acute deterioration of placental function that identifies the fetus at risk of failing to adapt to labour. Indeed, the risks factors for this pattern all impair placental function; however, further work is needed to understand the potential mechanisms. Although this pattern is not synchronised with contractions, it is rare antenatally, suggesting that uterine contractions, or changes in uterine tone, during labour are part of the mechanism. Hence, computerised spectral analysis of FHRV is an important but still developing area of FHR monitoring.^142^, ^143^ Future work should seek to examine whether patterns of increased FHRV occurring early and late during the process of fetal compromise can be distinguished by spectral analysis and other modern signal analysis techniques.

We believe there is already sufficient evidence to illustrate that increased FHRV observed in early labour can represent an early warning sign of impaired placental function, and additionally that increased FHRV in the setting of deep repetitive decelerations can indicate impending fetal compromise. An important corollary, and a significant departure from standard teaching, is that although suppression of FHRV is undoubtedly an ominous sign, the absence of suppressed FHRV cannot be relied upon to exclude fetal compromise. The specific features of increased FHRV, alone and combined with late decelerations, should be incorporated into clinical interpretation, electronic fetal monitoring guidelines and computerised algorithms to improve the performance of fetal surveillance during labour. The authors believe that the evidence presented in this review may improve fetal surveillance, enable timely conservative intrauterine resuscitation measures and recognition of loss of fetal compensatory reserve, and may improve the clinical decision making on intrapartum CTG recordings with episodes of increased FHRV. Standardisation of the terminology surrounding increased FHRV will likely improve the uptake of this knowledge.

## AUTHOR CONTRIBUTIONs

MJT conceived this review. MJT and CAL undertook the publication search. MJT prepared the first draft of the manuscript. MJT, CAL, SA, AJG and KAT contributed to interpreting the findings, editing and critically revising the manuscript, approved the final version of the manuscript and have agreed to be accountable for all aspects of the work. All persons who qualify for authorship are listed and all persons designated as authors, qualify for authorship.

## CONFLICT OF INTERESTS

Open access funding provided by University of Helsinki including Helsinki University Central Hospital. Mikko Tarvonen has received support from the Foundation for Paediatric Research, Finska Läkaresällskapet and Olga & Vilho Linnamo Foundation. Sture Andersson has received grants from a Special Governmental Subsidy for Clinical Research, Finska Läkaresällskapet, and the Society for Paediatric Research in Finland. Christopher Lear and Alistair Gunn received funding for this review from the Health Research Council of New Zealand. The sponsors had no role in the study design; collection, analysis, or interpretation of data, writing of the report, or in the decision to submit the report for publication. The authors have no interests to declare. Completed disclosure of interest forms are available to view online as supporting information.

## ETHICAL APPROVAL

No human or animal subjects were involved in this manuscript.

## Supporting information


Appendix S1

## Data Availability

The data that support the findings of this study are available on request from the corresponding author. The data are not publicly available due to privacy or ethical restrictions.

## References

[1] Nelson KB , Dambrosia JM , Ting TY , Grether JK . Uncertain value of electronic fetal monitoring in predicting cerebral palsy. N Engl J Med. 1996;334:613–8. 8592523 10.1056/NEJM199603073341001

[2] Low JA , Victory R , Derrick EJ . Predictive value of electronic fetal monitoring for intrapartum fetal asphyxia with metabolic acidosis. Obstet Gynecol. 1999;93:285–91. 9932571 10.1016/s0029-7844(98)00441-4

[3] Vintzileos AM , Smulian JC . Decelerations, tachycardia, and decreased variability: have we overlooked the significance of longitudinal fetal heart rate changes for detecting intrapartum fetal hypoxia? Am J Obstet Gynecol. 2016;215:261–4. 27568857 10.1016/j.ajog.2016.05.046

[4] Parer JT , Ugwumadu A . Impediments to a unified international approach to the interpretation and management of intrapartum cardiotocographs. J Matern Fetal Neonatal Med. 2017;30:272–3. 27046650 10.3109/14767058.2016.1174207

[5] Alfirevic Z , Devane D , Gyte GM , Cuthbert A . Continuous cardiotocography (CTG) as a form of electronic fetal monitoring (EFM) for fetal assessment during labour. Cochrane Database Syst Rev. 2017;2:CD006066. 16856111 10.1002/14651858.CD006066

[6] Ugwumadu A . Are we (mis)guided by current guidelines on intrapartum fetal heart rate monitoring? Case for a more physiological approach to interpretation. BJOG. 2014;121:1063–70. 24920154 10.1111/1471-0528.12900

[7] Garabedian C , De Jonckheere J , Butruille L , Deruelle P , Storme L , Houfflin‐Debarge V . Understanding fetal physiology and second line monitoring during labor. J Gynecol Obstet Hum Reprod. 2017;46:113–7. 28403965 10.1016/j.jogoh.2016.11.005

[8] O’Brien‐Abel N . Clinical implications of fetal heart rate interpretation based on underlying physiology. MCN Am J Matern Child Nurs. 2020;45:82–91. 31714283 10.1097/NMC.0000000000000596

[9] Jia YJ , Chen X , Cui HY , Whelehan V , Archer A , Chandraharan E . Physiological CTG interpretation: the significance of baseline fetal heart rate changes after the onset of decelerations and associated perinatal outcomes. J Matern Fetal Neonatal Med. 2021;34:2349–54. 31533502 10.1080/14767058.2019.1666819

[10] Parer JT , Ikeda T . A framework for standardized management of intrapartum fetal heart rate patterns. Am J Obstet Gynecol. 2007;197(26):e1–6. 10.1016/j.ajog.2007.03.037 17618744

[11] Turner JM , Mitchell MD , Kumar SS . The physiology of intrapartum fetal compromise at term. Am J Obstet Gynecol. 2020;222:17–26. 31351061 10.1016/j.ajog.2019.07.032

[12] Lear CA , Westgate JA , Ugwumadu A , Nijhuis JG , Stone PR , Georgieva A , et al. Understanding fetal heart rate patterns that may predict antenatal and intrapartum neural injury. Semin Pediatr Neurol. 2018;28:3–16. 30522726 10.1016/j.spen.2018.05.002

[13] Georgieva A , Lear CA , Westgate JA , Kasai M , Miyagi E , Ikeda T , et al. Deceleration area and capacity during labour‐like umbilical cord occlusions identify evolving hypotension: a controlled study in fetal sheep. BJOG. 2021;128:1433–42. 33369871 10.1111/1471-0528.16638

[14] Ghi T , Di Pasquo E , Dall’Asta A , Commare A , Melandri E , Casciaro A , et al. Intrapartum fetal heart rate between 150 and 160 bpm at or after 40 weeks and labor outcome. Acta Obstet Gynecol Scand. 2021;100:548–54. 33051873 10.1111/aogs.14024

[15] Lepercq J , Nghiem MA , Goffinet F . Fetal heart rate nadir during bradycardia and umbilical artery acidemia at birth. Acta Obstet Gynecol Scand. 2021;100:964–70. 33314025 10.1111/aogs.14061

[16] Paul RH , Suidan AK , Yeh S , Schifrin BS , Hon EH . Clinical fetal monitoring. VII. The evaluation and significance of intrapartum baseline FHR variability. Am J Obstet Gynecol. 1975;123:206–10. 1172373

[17] Willcourt RJ , King JC , Indyk L , Queenan JT . The relationship of fetal heart rate patterns to the fetal transcutaneous PO2. Am J Obstet Gynecol. 1981;140:760–9. 7258257 10.1016/0002-9378(81)90737-7

[18] Parer JT , King T , Flanders S , Fox M , Kilpatrick SJ . Fetal acidemia and electronic fetal heart rate patterns: is there evidence of an association? J Matern Fetal Neonatal Med. 2006;19:289–94. 16753769 10.1080/14767050500526172

[19] Holzmann M , Wretler S , Cnattingius S , Nordström L . Cardiotocography patterns and risk of intrapartum fetal acidemia. J Perinat Med. 2015;43:473–9. 24914710 10.1515/jpm-2014-0105

[20] Garabedian C , Champion C , Servan‐Schreiber E , Butruille L , Aubry E , Sharma D , et al. A new analysis of heart rate variability in the assessment of fetal parasympathetic activity: an experimental study in a fetal sheep model. PLoS One. 2017;12:e0180653. 28700617 10.1371/journal.pone.0180653 PMC5503275

[21] Thaler I , Timor‐Tritsch IE , Blumenfeld Z . Effect of acute hypoxia on human fetal heart rate. The significance of increased heart rate variability. Acta Obstet Gynecol Scand. 1985;64:47–50. 3976376 10.3109/00016348509154687

[22] Kurahashi H , Okumura A , Kubota T , Kidokoro H , Maruyama K , Hayakawa M , et al. Increased fetal heart rate variability in periventricular leukomalacia. Brain Dev. 2016;38:196–203. 26338690 10.1016/j.braindev.2015.08.008

[23] Westgate JA , Bennet L , Gunn AJ . Fetal heart rate variability changes during brief repeated umbilical cord occlusion in near term fetal sheep. Br J Obstet Gynaecol. 1999;106:664–71. 10428522 10.1111/j.1471-0528.1999.tb08365.x

[24] Durosier LD , Green G , Batkin I , Seely AJ , Ross MG , Richardson BS , et al. Sampling rate of heart rate variability impacts the ability to detect acidemia in ovine fetuses near‐term. Front Pediatr. 2014;2:38. 24829897 10.3389/fped.2014.00038 PMC4017161

[25] Ghesquière L , De Jonckheere J , Drumez E , Sharma D , Aubry E , Deruelle P , et al. Parasympathetic nervous system response to acidosis: evaluation in an experimental fetal sheep model. Acta Obstet Gynecol Scand. 2019;98:433–9. 30566227 10.1111/aogs.13515

[26] Liu L , Tuuli MG , Roehl KA , Odibo AO , Macones GA , Cahill AG . Electronic fetal monitoring patterns associated with respiratory morbidity in term neonates. Am J Obstet Gynecol. 2015;213:681.e1–6. 10.1016/j.ajog.2015.07.013 PMC5454805 26193688

[27] Polnaszek B , López JD , Clark R , Raghuraman N , Macones GA , Cahill AG . Marked variability in intrapartum electronic fetal heart rate patterns: association with neonatal morbidity and abnormal arterial cord gas. J Perinatol. 2020;40:56–62. 31578422 10.1038/s41372-019-0520-9 PMC7202403

[28] Gracia‐Perez‐Bonfils A , Vigneswaran K , Cuadras D , Chandraharan E . Does the saltatory pattern on cardiotocograph (CTG) trace really exist? The ZigZag pattern as an alternative definition and its correlation with perinatal outcomes. J Matern Fetal Neonatal Med. 2021;34:3537–45. 31722586 10.1080/14767058.2019.1686475

[29] Tarvonen M , Hovi P , Sainio S , Vuorela P , Andersson S , Teramo K . Intrapartum zigzag pattern of fetal heart rate is an early sign of fetal hypoxia: a large obstetric retrospective cohort study. Acta Obstet Gynecol Scand. 2021;100:252–62. 32981037 10.1111/aogs.14007 PMC7894352

[30] Tarvonen M , Hovi P , Sainio S , Vuorela P , Andersson S , Teramo K . Intrapartal cardiotocographic patterns and hypoxia‐related perinatal outcomes in pregnancies complicated by gestational diabetes mellitus. Acta Diabetol. 2021;58:1563–73. 34151398 10.1007/s00592-021-01756-0 PMC8505288

[31] Macones GA , Hankins GD , Spong CY , Hauth J , Moore T . The 2008 National Institute of Child Health and Human Development workshop report on electronic fetal monitoring: update on definitions, interpretation, and research guidelines. Obstet Gynecol. 2008;112:661–6. 18757666 10.1097/AOG.0b013e3181841395

[32] Ayres‐de‐Campos D , Spong CY , Chandraharan E . For the FIGO intrapartum fetal monitoring expert consensus panel. FIGO consensus guidelines on intrapartum fetal monitoring: cardiotocography. Int J Gynecol Obstet. 2015;131:13–24. 10.1016/j.ijgo.2015.06.020 26433401

[33] NICE. National Institute for Health and Care Excellence . Addendum to intrapartum care: care for healthy women and babies. Clinical guideline [CG190.1]; 2017. Methods, evidence and recommendations. Final version. Pages: 136–7, 141–2. Available from: www.nice.org.uk/guidance/cg190/evidence/addendum‐190.1‐pdf‐4365472285

[34] Gimovsky ML , Goh W , Fitzgerald K . Fetal monitoring casebook. The saltatory fetal heart rate pattern. J Perinatol. 1991;11:386–9. 1770399

[35] Westgate JA , Bennet L , Gunn AJ . Fetal seizures causing increased heart rate variability during terminal fetal hypoxia. Am J Obstet Gynecol. 1999;181:765–6. 10486503 10.1016/s0002-9378(99)70532-6

[36] Yanamandra N , Chandraharan E . Saltatory and sinusoidal fetal heart rate patterns and significance of FHR’overshoots. Curr Women’s Health Rev. 2013;9:175–82.

[37] Lear CA , Galinsky R , Wassink G , Mitchell CJ , Davidson JO , Westgate JA , et al. Sympathetic neural activation does not mediate heart rate variability during repeated brief umbilical cord occlusions in near‐term fetal sheep. J Physiol. 2016;594:1265–77. 25864517 10.1113/JP270125 PMC4771778

[38] Lear CA , Westgate JA , Kasai M , Beacom MJ , Maeda Y , Magawa S , et al. Parasympathetic activity is the key regulator of heart rate variability between decelerations during brief repeated umbilical cord occlusions in fetal sheep. Am J Physiol Regul Integr Comp Physiol. 2020;319:R541–50. 32877241 10.1152/ajpregu.00186.2020

[39] Kühnert M , Schmidt S . 24 hour‐CTG monitoring: comparison of normal pregnancies and pregnancies with placenta insufficiency. J Perinat Med. 2001;29:42–54. 11234616 10.1515/JPM.2001.006

[40] Xie W , Archer A , Li C , Cui H , Chandraharan E . Fetal heart rate changes observed on the CTG trace during instrumental vaginal delivery. J Matern Fetal Neonatal Med. 2017;10:1–8. 10.1080/14767058.2017.1373084 28851252

[41] Tarvonen M , Sainio S , Hämäläinen E , Hiilesmaa V , Andersson S , Teramo K . Saltatory pattern of fetal heart rate during labor is a sign of fetal hypoxia. Neonatology. 2020;117:111–7. 31846958 10.1159/000504941

[42] Tarvonen M , Hovi P , Sainio S , Vuorela P , Andersson S , Teramo K . Factors associated with intrapartum ZigZag pattern of fetal heart rate: a retrospective one‐year cohort study of 5150 singleton childbirths. Eur J Obstet Gynecol Reprod Biol. 2021;258:118–25. 33421808 10.1016/j.ejogrb.2020.12.056

[43] Parer JT , Dijkstra HR , Vredebregt PP , Harris JL , Krueger TR , Reuss ML . Increased fetal heart rate variability with acute hypoxia in chronically instrumented sheep. Eur J Obstet Gynecol Reprod Biol. 1980;10:393–9. 7190938 10.1016/0028-2243(80)90025-8

[44] O’Brien‐Abel NE , Benedetti TJ . Saltatory fetal heart rate pattern. J Perinatol. 1992;1:13–7. 1560284

[45] Cahill AG , Roehl KA , Odibo AO , Macones GA . Association and prediction of neonatal acidemia. Am J Obstet Gynecol. 2012;207:206.e1–8. 10.1016/j.ajog.2012.06.046 22939728

[46] Cahill AG , Tuuli MG , Stout MJ , López JD , Macones GA . A prospective cohort study of fetal heart rate monitoring: deceleration area is predictive of fetal acidemia. Am J Obstet Gynecol. 2018;218:523.e1–e12. 10.1016/j.ajog.2018.01.026 PMC5916338 29408586

[47] George S , Gunn AJ , Westgate JA , Brabyn C , Guan J , Bennet L . Fetal heart rate variability and brain stem injury after asphyxia in preterm fetal sheep. Am J Physiol Regul Integr Comp Physiol. 2004;287:R925–33. 15191906 10.1152/ajpregu.00263.2004

[48] Yamaguchi K , Lear CA , Beacom MJ , Ikeda T , Gunn AJ , Bennet L . Evolving changes in fetal heart rate variability and brain injury after hypoxia‐ischaemia in preterm fetal sheep. J Physiol. 2018;596:6093–104. 29315570 10.1113/JP275434 PMC6265593

[49] Peebles DM , Spencer JA , Edwards AD , Wyatt JS , Reynolds EO , Cope M , et al. Relation between frequency of uterine contractions and human fetal cerebral oxygen saturation studied during labour by near infrared spectroscopy. Br J Obstet Gynaecol. 1994;101(1):44–8. 8297867 10.1111/j.1471-0528.1994.tb13008.x

[50] Ross MG , Gala R . Use of umbilical artery base excess: algorithm for the timing of hypoxic injury. Am J Obstet Gynecol. 2002;187:1–9. 12114881 10.1067/mob.2002.123204

[51] Wiberg N , Kallen K , Herbst A , Olofsson P . Relation between umbilical cord blood pH, base deficit, lactate, 5‐minute Apgar score and development of hypoxic ischemic encephalopathy. Acta Obstet Gynecol Scand. 2010;89:1263–9. 20846059 10.3109/00016349.2010.513426

[52] Kikuchi H , Noda S , Katsuragi S , Ikeda T , Horio H . Evaluation of 3‐tier and 5‐tier FHR pattern classifications using umbilical blood pH and base excess at delivery. PLoS One. 2020;15:e0228630. 32027690 10.1371/journal.pone.0228630 PMC7004356

[53] Giussani DA . The fetal brain sparing response to hypoxia: physiological mechanisms. J Physiol. 2016;594:1215–30. 26496004 10.1113/JP271099 PMC4721497

[54] Jensen A , Garnier Y , Berger R . Dynamics of fetal circulatory responses to hypoxia and asphyxia. Eur J Obstet Gynecol Reprod Biol. 1999;84:155–72. 10428339 10.1016/s0301-2115(98)00325-x

[55] Kiserud T , Rasmussen S , Skulstad S . Blood flow and the degree of shunting through the ductus venosus in the human fetus. Am J Obstet Gynecol. 2000;182:147–53. 10649170 10.1016/s0002-9378(00)70504-7

[56] Ferrazzi E , Bozzo M , Rigano S , Bellotti M , Morabito A , Pardi G , et al. Temporal sequence of abnormal doppler changes in the peripheral and central circulatory systems of the severely growth‐restricted fetus. Ultrasound Obstet Gynecol. 2002;19:140–6. 11876805 10.1046/j.0960-7692.2002.00627.x

[57] Lear CA , Galinsky R , Wassink G , Yamaguchi K , Davidson JO , Westgate JA , et al. The myths and physiology surrounding intrapartum decelerations: the critical role of the peripheral chemoreflex. J Physiol. 2016;594:4711–25. 27328617 10.1113/JP271205 PMC5009777

[58] Tarvonen M . Are we paying enough attention to uterine contractions? BJOG. 2022. 10.1111/1471-0528.17153 35304970

[59] Low JA . Intrapartum fetal asphyxia: definition, diagnosis, and classification. Am J Obstet Gynecol. 1997;176:957–9. 9166151 10.1016/s0002-9378(97)70385-5

[60] Gluckman PD , Pinal CS , Gunn AJ . Hypoxic‐ischemic brain injury in the newborn: pathophysiology and potential strategies for intervention. Semin Neonatol. 2001;6:109–20. 11483017 10.1053/siny.2001.0042

[61] Hayes BC , McGarvey C , Mulvany S , Kennedy J , Geary MP , Matthews TG , et al. A case‐control study of hypoxic‐ischemic encephalopathy in newborn infants at >36 weeks gestation. Am J Obstet Gynecol. 2013;209:29.e1–29.e19. 10.1016/j.ajog.2013.03.023 23524176

[62] Jonsson M , Ågren J , Nordén‐Lindeberg S , Ohlin A , Hanson U . Neonatal encephalopathy and the association to asphyxia in labor. Am J Obstet Gynecol. 2014;211(667):e1–8. 10.1016/j.ajog.2014.06.027 24949542

[63] Morton SU , Brodsky D . Fetal physiology and the transition to extrauterine life. Clin Perinatol. 2016;43:395–407. 27524443 10.1016/j.clp.2016.04.001 PMC4987541

[64] Dhillon SK , Lear CA , Galinsky R , Wassink G , Davidson JO , Juul S , et al. The fetus at the tipping point: modifying the outcome of fetal asphyxia. J Physiol. 2018;596:5571–92. 29774532 10.1113/JP274949 PMC6265539

[65] Gunn AJ , Parer JT , Mallard EC , Williams CE , Gluckman PD . Cerebral histologic and electrocorticographic changes after asphyxia in fetal sheep. Pediatr Res. 1992;31:486–91. 1603625 10.1203/00006450-199205000-00016

[66] Gunn AJ , Maxwell L , De Haan HH , Bennet L , Williams CE , Gluckman PD , et al. Delayed hypotension and subendocardial injury after repeated umbilical cord occlusion in near‐term fetal lambs. Am J Obstet Gynecol. 2000;183:1564–72. 11120529 10.1067/mob.2000.108084

[67] Graham EM , Petersen SM , Christo DK , Fox HE . Intrapartum electronic fetal heart rate monitoring and the prevention of perinatal brain injury. Obstet Gynecol. 2006;108:656–66. 16946228 10.1097/01.AOG.0000230533.62760.ef

[68] Polglase GR , Ong T , Hillman NH . Cardiovascular alterations and multiorgan dysfunction after birth asphyxia. Clin Perinatol. 2016;43:469–83. 27524448 10.1016/j.clp.2016.04.006 PMC4988334

[69] Dall’Asta A , Kumar S . Prelabor and intrapartum doppler ultrasound to predict fetal compromise. Am J Obstet Gynecol MFM. 2021;5:100479. 10.1016/j.ajogmf.2021.100479 34496306

[70] Gold N , Herry CL , Wang X , Frasch MG . Fetal cardiovascular decompensation during labor predicted from the individual heart rate tracing: a machine learning approach in near‐term fetal sheep model. Front Pediatr. 2021;9:593889. 34026680 10.3389/fped.2021.593889 PMC8132964

[71] Lear CA , Westgate JA , Bennet L , Ugwumadu A , Stone PR , Tournier A , et al. Fetal defenses against intrapartum head compression‐implications for intrapartum decelerations and hypoxic‐ischemic injury. Am J Obstet Gynecol. 2021:S0002‐9378(21)02581‐3. 10.1016/j.ajog.2021.11.1352 34801443

[72] King T , Parer J . The physiology of fetal heart rate patterns and perinatal asphyxia. J Perinat Neonatal Nurs. 2000;14:19–39. quiz 102–3. 11930377 10.1097/00005237-200012000-00003

[73] Frey HA , Liu X , Lynch CD , Musindi W , Samuels P , Rood KM , et al. An evaluation of fetal heart rate characteristics associated with neonatal encephalopathy: a case‐control study. BJOG. 2018;125:1480–7. 29575562 10.1111/1471-0528.15222

[74] Bennet L , Westgate JA , Liu YC , Wassink G , Gunn AJ . Fetal acidosis and hypotension during repeated umbilical cord occlusions are associated with enhanced chemoreflex responses in near‐term fetal sheep. J Appl Physiol. 1985;2005(99):1477–82. 10.1152/japplphysiol.00431.2005 15976361

[75] Romano M , Bifulco P , Cesarelli M , Sansone M , Bracale M . Foetal heart rate power spectrum response to uterine contraction. Med Biol Eng Comput. 2006;44:188–201. 16937160 10.1007/s11517-006-0022-8

[76] Annunziata ML , Tagliaferri S , Esposito FG , Giuliano N , Mereghini F , Di Lieto A , et al. Computerized analysis of fetal heart rate variability signal during the stages of labor. J Obstet Gynaecol Res. 2016;42:258–65. 26787219 10.1111/jog.12908

[77] Brotanek V , Scheffs J . The pathogenesis and significance of saltatory patterns in the fetal heart rate. Int J Gynaecol Obstet. 1973;11:223–8.

[78] Dalton KJ , Dawes GS , Patrick JE . Diurnal, respiratory, and other rhythms of fetal heart rate in lambs. Am J Obstet Gynecol. 1977;127:414–24. 556883 10.1016/0002-9378(77)90500-2

[79] Dalton KJ , Dawes GS , Patrick JE . The autonomic nervous system and fetal heart rate variability. Am J Obstet Gynecol. 1983;146:456–62. 6859165 10.1016/0002-9378(83)90828-1

[80] Giussani DA , Unno N , Jenkins SL , Wentworth RA , Derks JB , Collins JH , et al. Dynamics of cardiovascular responses to repeated partial umbilical cord compression in late‐gestation sheep fetus. Am J Physiol. 1997;273:H2351–60. 9374772 10.1152/ajpheart.1997.273.5.H2351

[81] Ikeda T , Murata Y , Quilligan EJ , Parer JT , Theunissen IM , Cifuentes P , Doi S , Park SD . Fetal heart rate patterns in postasphyxiated fetal lambs with brain damage. Am J Obstet Gynecol 1998;179:1329–37, 1337. 9822525 10.1016/s0002-9378(98)70156-5

[82] Frasch MG , Herry CL , Niu Y , Giussani DA . First evidence that intrinsic fetal heart rate variability exists and is affected by hypoxic pregnancy. J Physiol. 2020;598:249–63. 31802494 10.1113/JP278773

[83] Lantto J , Erkinaro T , Haapsamo M , Huhta H , Alanne L , Kokki M , et al. Peripheral chemoreflex activation and cardiac function during hypoxemia in near term fetal sheep without placental compromise. J Appl Physiol (1985). 2021;131:1486–95. 10.1152/japplphysiol.01111.2020 34590908

[84] Yu ZY , Lumbers ER , Gibson KJ , Stevens AD . Effects on hypoxaemia on foetal heart rate, variability and cardiac rhythm. Clin Exp Pharmacol Physiol. 1998;25:577–84. 9673432 10.1111/j.1440-1681.1998.tb02255.x

[85] Kozuma S , Watanabe T , Bennet L , Green LR , Hanson MA . The effect of carotid sinus denervation on fetal heart rate variation in normoxia, hypoxia and post‐hypoxia in fetal sheep. Br J Obstet Gynaecol. 1997;104:460–5. 9141583 10.1111/j.1471-0528.1997.tb11498.x

[86] Lear CA , Davidson JO , Dhillon SK , King VJ , Lear BA , Magawa S , et al. Effects of antenatal dexamethasone and hyperglycemia on cardiovascular adaptation to asphyxia in preterm fetal sheep. Am J Physiol Regul Integr Comp Physiol. 2020;319:R653–65. 33074015 10.1152/ajpregu.00216.2020

[87] Martin CB Jr , Murata Y , Petrie RH , Parer JT . Respiratory movements in fetal rhesus monkeys. Am J Obstet Gynecol. 1974;119:939–48. 4366794 10.1016/0002-9378(74)90011-8

[88] Ikenoue T , Martin CB Jr , Murata Y , Ettinger BB , Lu PS . Effect of acute hypoxemia and respiratory acidosis on the fetal heart rate in monkeys. Am J Obstet Gynecol. 1981;141:797–806. 6797302 10.1016/0002-9378(81)90707-9

[89] Lear CA , Wassink G , Westgate JA , Nijhuis JG , Ugwumadu A , Galinsky R , et al. The peripheral chemoreflex: indefatigable guardian of fetal physiological adaptation to labour. J Physiol. 2018;596:5611–23. 29604081 10.1113/JP274937 PMC6265558

[90] Lear CA , Kasai M , Booth LC , Drury PP , Davidson JO , Maeda Y , et al. Peripheral chemoreflex control of fetal heart rate decelerations overwhelms the baroreflex during brief umbilical cord occlusions in fetal sheep. J Physiol. 2020;598:4523–36. 32705685 10.1113/JP279573

[91] Vanspranghels R , De Jonckheere J , Drumez E , Lauriot Dit Prevost A , Sharma D , Ghesquiere L , et al. Autonomic response to fetal acidosis using an experimental sheep model. Eur J Obstet Gynecol Reprod Biol. 2020;246:151–5. 32028142 10.1016/j.ejogrb.2020.01.018

[92] Westgate JA , Bennet L , de Haan HH , Gunn AJ . Fetal heart rate overshoot during repeated umbilical cord occlusion in sheep. Obstet Gynecol. 2001;97:454–9. 11239656 10.1016/s0029-7844(00)01123-6

[93] Papaioannou VE , Verkerk AO , Amin AS , de Bakker JM . Intracardiac origin of heart rate variability, pacemaker funny current and their possible association with critical illness. Curr Cardiol Rev. 2013;9:82–96. 22920474 10.2174/157340313805076359 PMC3584310

[94] Rosén KG , Dagbjartsson A , Henriksson BA , Lagercrantz H , Kjellmer I . The relationship between circulating catecholamines and ST waveform in the fetal lamb electrocardiogram during hypoxia. Am J Obstet Gynecol. 1984;149:190–5. 6720798 10.1016/0002-9378(84)90197-2

[95] Nunes I , Ayres‐de‐Campos D , Kwee A , Rosen KG . Prolonged saltatory fetal heart rate pattern leading to newborn metabolic acidosis. Clin Exp Obstet Gynecol. 2014;41:507–11. 25864248

[96] Tournier A , Beacom M , Westgate JA , Bennet L , Garabedian C , Ugwumadu A , et al. Physiological control of fetal heart rate variability during labour: implications and controversies. J Physiol. 2022;600:431–50. 34951476 10.1113/JP282276

[97] Lear CA , Beacom MJ , Kasai M , Westgate JA , Galinsky R , Magawa S , Miyagi E , Ikeda T , Bennet L , Gunn AJ . Circulating catecholamines partially regulate T‐wave morphology but not heart rate variability during repeated umbilical cord occlusions in fetal sheep. 10.1152/ajpregu.00026.2020 32491938

[98] Magawa S , Ikeda T , Bennet L , Gunn AJ . Effects of β‐adrenergic stimulation on fetal heart rate, heart rate variability, and T‐wave elevation during brief umbilical cord occlusions in fetal sheep. Am J Physiol Regul Integr Comp Physiol. 2020;319:R551–9. 32877238 10.1152/ajpregu.00221.2020

[99] Galinsky R , Jensen EC , Bennet L , Mitchell CJ , Gunn ER , Wassink G , et al. Sustained sympathetic nervous system support of arterial blood pressure during repeated brief umbilical cord occlusions in near‐term fetal sheep. Am J Physiol Regul Integr Comp Physiol. 2014;306:R787–95. 24647590 10.1152/ajpregu.00001.2014

[100] Hon EH , Lee ST . Electronic evaluation of the fetal heart rate. Viii. Patterns preceding fetal death, further observations. Am J Obstet Gynecol. 1963;87:814–26. 14085784

[101] Caldeyro‐Barcia R , Casacuberta C , Bustos C , Giussi G , Gulin L , Escarcena L , et al. Correlation of intrapatum changes in fetal hcart rate with fetal blood oxygen and acid base balance. In: Adamsons K , editor. Diagnosis and treatment fetal disorders. Berlin‐Heidelberg‐New York: Springer; 1968.

[102] Hammacher K , Hüter KA , Bokelmann J , Werners PH . Foetal heart frequency and perinatal condition of the foetus and newborn. Gynaecologia. 1968;166:349–60. 5699016 10.1159/000302346

[103] Lu K , Holzmann M , Abtahi F , Lindecrantz K , Lindqvist PG , Nordstrom L . Fetal heart rate short term variation during labor in relation to scalp blood lactate concentration. Acta Obstet Gynecol Scand. 2018;97:1274–80. 29799630 10.1111/aogs.13390

[104] Teramo KA , Widness JA , Clemons GK , Voutilainen P , McKinlay S , Schwartz R . Amniotic fluid erythropoietin correlates with umbilical plasma erythropoietin in normal and abnormal pregnancy. Obstet Gynecol. 1987;69:710–6. 3574798

[105] Kakuya F , Shirai M , Takase M , Ishii N , Ishioka T , Hayashi T , et al. Relationship between erythropoietin levels both in cord serum and amniotic fluid at birth and abnormal fetal heart rate records. Pediatr Int. 2002;44:414–9. 12139568 10.1046/j.1442-200x.2002.01583.x

[106] Teramo K , Kari MA , Eronen M , Markkanen H , Hiilesmaa V . High amniotic fluid erythropoietin levels are associated with an increased frequency of fetal and neonatal morbidity in type 1 diabetic pregnancies. Diabetologia. 2004;47:1695–703. 15502930 10.1007/s00125-004-1515-3

[107] Escobar J , Teramo K , Stefanovic V , Andersson S , Asensi MA , Arduini A . Amniotic fluid oxidative and nitrosative stress biomarkers correlate with fetal chronic hypoxia in diabetic pregnancies. Neonatology. 2013;103:193–8. 23295371 10.1159/000345194

[108] Widness JA , Teramo KA , Clemons GK , Coustan DR , Cavalieri RL , Oh W , et al. Correlation of the interpretation of fetal heart rate records with cord plasma erythropoietin levels. Br J Obstet Gynaecol. 1985;92:326–32. 3986165 10.1111/j.1471-0528.1985.tb01104.x

[109] Kitanaka T , Alonso JG , Gilbert RD , Siu BL , Clemons GK , Longo LD . Fetal responses to long‐term hypoxemia in sheep. Am J Physiol. 1989;256:R1348–54. 2500037 10.1152/ajpregu.1989.256.6.R1348

[110] Cibils LA . Clinical significance of fetal heart rate patterns during labor. II. Late decelerations. Am J Obstet Gynecol. 1975;123:473–94. 1180296 10.1016/0002-9378(75)90035-6

[111] Jazayeri A , Tsibris JC , Spellacy WN . Elevated umbilical cord plasma erythropoietin levels in prolonged pregnancies. Obstet Gynecol. 1998;92:61–3. 9649094 10.1016/s0029-7844(98)00126-4

[112] Eskild A , Haavaldsen C , Vatten LJ . Placental weight and placental weight to birthweight ratio in relation to Apgar score at birth: a population study of 522 360 singleton pregnancies. Acta Obstet Gynecol Scand. 2014;93:1302–8. 25244579 10.1111/aogs.12509

[113] Eskild A , Strøm‐Roum EM , Haavaldsen C . Does the biological response to fetal hypoxia involve angiogenesis, placental enlargement and preeclampsia? Paediatr Perinat Epidemiol. 2016;30:305–9. 27038011 10.1111/ppe.12283 PMC4825407

[114] Radaelli T , Varastehpour A , Catalano P , Hauguel‐de Mouzon S . Gestational diabetes induces placental genes for chronic stress and inflammatory pathways. Diabetes. 2003;52:2951–8. 14633856 10.2337/diabetes.52.12.2951

[115] Desoye G , Hauguel‐de MS . The human placenta in gestational diabetes mellitus. The insulin and cytokine network. Diabetes Care. 2007;30(Suppl 2):S120–6. 17596459 10.2337/dc07-s203

[116] Blad S , Welin AK , Kjellmer I , Rosén KG , Mallard C . ECG and heart rate variability changes in preterm and near‐term fetal lamb following LPS exposure. Reprod Sci. 2008;15:572–83. 18456675 10.1177/1933719107314060

[117] Lear CA , Davidson JO , Galinsky R , Yuill CA , Wassink G , Booth LC , et al. Subclinical decelerations during developing hypotension in preterm fetal sheep after acute on chronic lipopolysaccharide exposure. Sci Rep. 2015;5:16201. 26537688 10.1038/srep16201 PMC4633652

[118] Magawa S , Lear CA , Beacom MJ , King VJ , Kasai M , Galinsky R , et al. Fetal heart rate variability is a biomarker of rapid but not progressive exacerbation of inflammation in preterm fetal sheep. Sci Rep. 2022;12(1):1771. 35110628 10.1038/s41598-022-05799-3 PMC8810879

[119] Sinaci S , Ocal DF , Ozden Tokalioglu E , Halici Ozturk F , Aydin Senel S , Keskin LH , et al. Cardiotocographic features in COVID‐19 infected pregnant women. J Perinat Med. 2021;50:46–55. 10.1515/jpm-2021-0132 34411469

[120] Gracia‐Perez‐Bonfils A , Martinez‐Perez O , Llurba E , Chandraharan E . Fetal heart rate changes on the cardiotocograph trace secondary to maternal COVID‐19 infection. Eur J Obstet Gynecol Reprod Biol. 2020;252:286–93. 32645644 10.1016/j.ejogrb.2020.06.049 PMC7331544

[121] Galli L , Dall’Asta A , Whelehan V , Archer A , Chandraharan E . Intrapartum cardiotocography patterns observed in suspected clinical and subclinical chorioamnionitis in term fetuses. J Obstet Gynaecol Res. 2019;45:2343–50. 31621146 10.1111/jog.14133

[122] Sukumaran S , Pereira V , Mallur S , Chandraharan E . Cardiotocograph (CTG) changes and maternal and neonatal outcomes in chorioamnionitis and/or funisitis confirmed on histopathology. Eur J Obstet Gynecol Reprod Biol. 2021;260:183–8. 33838555 10.1016/j.ejogrb.2021.03.029

[123] Pereira S , Lau K , Modestini C , Wertheim D , Chandraharan E . Absence of fetal heart rate cycling on the intrapartum cardiotocograph (CTG) is associated with intrapartum pyrexia and lower Apgar scores. J Matern Fetal Neonatal Med. 2021;22:1–6. 10.1080/14767058.2021.1940130 34157928

[124] Robinson B . A review of NICHD standardized nomenclature for cardiotocography: the importance of speaking a common language when describing electronic fetal monitoring. Rev Obstet Gynecol. 2008;1:56–60. 18769667 PMC2505172

[125] Clark SL , Nageotte MP , Garite TJ , Freeman RK , Miller DA , Simpson KR , et al. Intrapartum management of category II fetal heart rate tracings: towards standardization of care. Am J Obstet Gynecol. 2013;209:89–97. 23628263 10.1016/j.ajog.2013.04.030

[126] Miller DA , Miller LA . Electronic fetal heart rate monitoring: applying principles of patient safety. Am J Obstet Gynecol. 2012;206:278–83. 22000897 10.1016/j.ajog.2011.08.016

[127] Robinson B , Nelson L . A review of the proceedings from the 2008 NICHD workshop on standardized nomenclature for cardiotocography: update on definitions, interpretative systems with management strategies, and research priorities in relation to intrapartum electronic fetal monitoring. Rev Obstet Gynecol. 2008;1:186–92. 19173023 PMC2621055

[128] Ayres‐de‐Campos D , Arulkumaran S . FIGO intrapartum fetal monitoring expert consensus panel. FIGO consensus guidelines on intrapartum fetal monitoring: physiology of fetal oxygenation and the main goals of intrapartum fetal monitoring. Int J Gynaecol Obstet. 2015;131:5–8. 26433399 10.1016/j.ijgo.2015.06.018

[129] ACOG American College of Obstetricians and Gynecologists. Practice bulletin no. 116: management of intrapartum fetal heart rate tracings. Obstet Gynecol. 2010;116:1232–40. 20966730 10.1097/AOG.0b013e3182004fa9

[130] NICHD . Electronic fetal heart rate monitoring: research guidelines for interpretation. National Institute of Child Health and Human Development research planning workshop. Am J Obstet Gynecol. 1997;177:1385–90. 9423739

[131] Kenepp N , Kumar S , Shelley W , Stanley C , Gabbe S , Gutsche B . Fetal and neonatal hazards of maternal hydration with 5% dextrose before caesarean section. Lancet. 1982;1:1150–2. 6122939 10.1016/s0140-6736(82)92227-9

[132] Philipps AF , Porte PJ , Stabinsky S , Rosenkrantz TS , Raye JR . Effects of chronic fetal hyperglycemia upon oxygen consumption in the ovine uterus and conceptus. J Clin Invest. 1984;74:279–86. 6429196 10.1172/JCI111412 PMC425210

[133] Teramo K , Piñeiro‐Ramos JD . Fetal chronic hypoxia and oxidative stress in diabetic pregnancy. Could fetal erythropoietin improve offspring outcomes? Free Radic Biol Med. 2019;142:32–7. 30898666 10.1016/j.freeradbiomed.2019.03.012

[134] Salvesen DR , Brudenell JM , Snijders RJ , Ireland RM , Nicolaides KH . Fetal plasma erythropoietin in pregnancies complicated by maternal diabetes mellitus. Am J Obstet Gynecol. 1993;168:88–94. 8420356 10.1016/s0002-9378(12)90891-1

[135] Salvesen DR , Freeman J , Brudenell JM , Nicolaides KH . Prediction of fetal acidaemia in pregnancies complicated by maternal diabetes mellitus by biophysical profile scoring and fetal heart rate monitoring. Br J Obstet Gynaecol. 1993;100:227–33. 8476827 10.1111/j.1471-0528.1993.tb15235.x

[136] Taricco E , Radaelli T , Rossi G , Nobile de Santis MS , Bulfamante GP , Avagliano L , et al. Effects of gestational diabetes on fetal oxygen and glucose levels in vivo. BJOG. 2009;116:1729–35. 19832834 10.1111/j.1471-0528.2009.02341.x

[137] Westgate JA , Wibbens B , Bennet L , Wassink G , Parer JT , Gunn AJ . The intrapartum deceleration in center stage: a physiologic approach to the interpretation of fetal heart rate changes in labor. Am J Obstet Gynecol. 2007;197(236):e1–11. 10.1016/j.ajog.2007.03.063 17826402

[138] Shaw CJ , Lees CC , Giussani DA . Variations on fetal heart rate variability. J Physiol. 2016;594:1279–80. 26926317 10.1113/JP270717 PMC4771802

[139] Altman DG , Andersen PK . Calculating the number needed to treat for trials where the outcome is time to an event. BMJ. 1999;319:1492–5. 10582940 10.1136/bmj.319.7223.1492 PMC1117211

[140] Downs T , Zlomke E . Fetal heart rate pattern notification guidelines and suggested management algorithm for intrapartum electronic fetal heart rate monitoring. Perm J. 2007;11:22–8. 21412478 10.7812/tpp/07-024 PMC3048435

[141] Reddy UM , Weiner SJ , Saade GR , Varner MW , Blackwell SC , Thorp JM Jr , et al. Eunice Kennedy Shriver National Institute of Child Health and Human Development (NICHD) maternal‐fetal medicine units (MFMU) network. Intrapartum resuscitation interventions for category II fetal heart rate tracings and improvement to category I. Obstet Gynecol. 2021;138:409–16. 34352857 10.1097/AOG.0000000000004508 PMC8506980

[142] Castro L , Loureiro M , Henriques TS , Nunes I . Systematic review of intrapartum fetal heart rate spectral analysis and an application in the detection of fetal acidemia. Front Pediatr. 2021;9:661400. 34408993 10.3389/fped.2021.661400 PMC8364976

[143] Zizzo AR , Kirkegaard I , Uldbjerg N , Hansen J , Mølgaard H . Towards better reliability in fetal heart rate variability using time domain and spectral domain analyses. A new method for assessing fetal neurological state? PLoS One. 2022;17:e0263272. 35231034 10.1371/journal.pone.0263272 PMC8887753

[144] Cibils LA . Clinical significance of fetal heart rate patterns during labor. I. Baseline patterns. Am J Obstet Gynecol. 1976;125:290–305. 1275022 10.1016/0002-9378(76)90563-9

