# Supplementary material for: Increased variability of fetal heart rate during labour: a review of preclinical and clinical studies
Source: BJOG. 2022 Jun 5;129(12):2070–81. doi: 10.1111/1471-0528.17234 (PMC9796294; doi:10.1111/1471-0528.17234)
Supplement: Supplementary file 1 — Appendix S1 [file BJO-129-2070-s001.docx]

**APPENDIX S1.** Gradual progress of the fetal hypoxia as changes in FHR recording during labour.


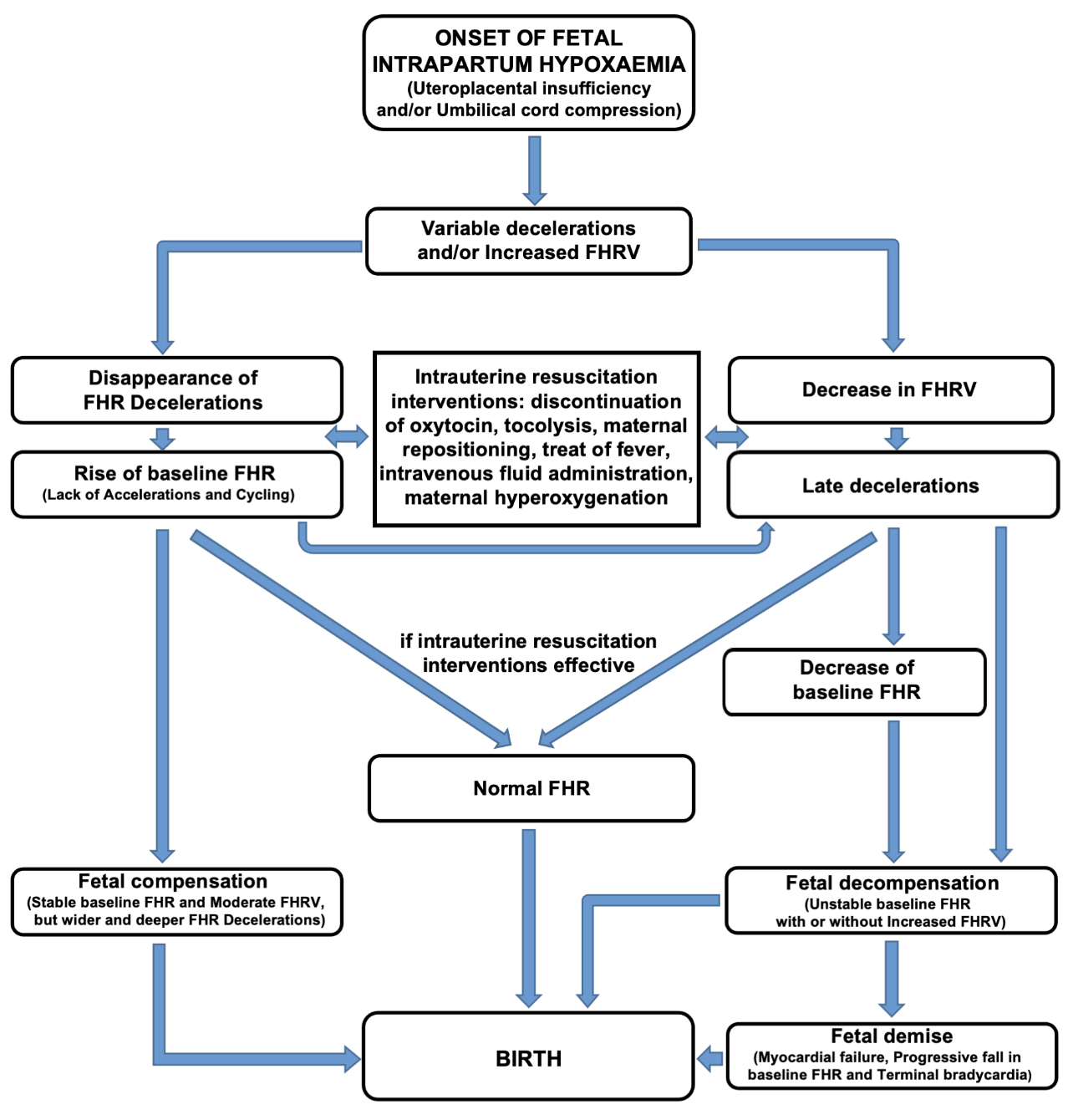


*FHR*, fetal heart rate. *FHRV*, fetal heart rate variability.

*Cycling*: A phenomenon of alternative active and quiescent periods, which occur in the CTG tracing as alternating episodes of reduced and normal FHRV; the occurrence of cycling indicates a non-depressed central nervous system.^123^
